# Dr John Blair Gibbs

**Published:** 1898-07

**Authors:** 


Dr John Blair Gibbs, assistant surgeon on duty with U. S. Marine Corps, was killed by the enemy at Guantanamo Bay early in the morning of June 12, 1898, while seeking the shelter of a block house during the fierce attack upon the camp by the Spaniards. He abandoned a lucrative practice to enter the service and was the first American officer killed on Cuban soil. His example is worthy of emulation and his early sacrifice is to be deeply lamented.

